# Bilingual Text4Walking Food Service Employee Intervention Pilot Study

**DOI:** 10.2196/mhealth.5328

**Published:** 2016-06-01

**Authors:** Susan Weber Buchholz, Diana Ingram, JoEllen Wilbur, Louis Fogg, Giselle Sandi, Angela Moss, Edith V Ocampo

**Affiliations:** ^1^ Rush University, College of Nursing Chicago, IL United States; ^2^ Rush University, Academic Affairs Chicago, IL United States

**Keywords:** text messaging, physical activity, workplace, employee, Spanish, feasibility, food service

## Abstract

**Background:**

Half of all adults in the United States do not meet the level of recommended aerobic physical activity. Physical activity interventions are now being conducted in the workplace. Accessible technology, in the form of widespread usage of cell phones and text messaging, is available for promoting physical activity.

**Objective:**

The purposes of this study, which was conducted in the workplace, were to determine (1) the feasibility of implementing a bilingual 12-week Text4Walking intervention and (2) the effect of the Text4Walking intervention on change in physical activity and health status in a food service employee population.

**Methods:**

Before conducting the study reported here, the Text4Walking research team developed a database of motivational physical activity text messages in English. Because Hispanic or Latino adults compose one-quarter of all adults employed in the food service industry, the Text4Walking team translated the physical activity text messages into Spanish. This pilot study was guided by the Physical Activity Health Promotion Framework and used a 1-group 12-week pre- and posttest design with food service employees who self-reported as being sedentary. The aim of the study was to increase the number of daily steps over the baseline by 3000 steps. Three physical activity text messages were delivered weekly. In addition, participants received 3 motivational calls during the study.

**Results:**

SPSS version 19.0 and R 3.0 were used to perform the data analysis. There were 33 employees who participated in the study (57.6% female), with a mean age of 43.7 years (SD 8.4). The study included 11 Hispanic or Latino participants, 8 of whom requested that the study be delivered in Spanish. There was a 100% retention rate in the study. At baseline, the participants walked 102 (SD 138) minutes/day (per self-report). This rate increased significantly (*P*=.008) to 182 (SD 219) minutes/day over the course of the study. The participants had a baseline mean of 10,416 (SD 5097) steps, which also increased significantly (*P*=.017) to 12,540 (SD 5149). They significantly improved their performance on their aerobic fitness test (*P*<.001). The participants had a baseline mean systolic blood pressure of 120 mm Hg and diastolic blood pressure of 76 mm Hg, a mean body mass index of 29.29 kg/m2, and a mean waist circumference of 36.95 inches, without significant changes seen at 12 weeks.

**Conclusions:**

We were able to conduct a motivational physical activity text messaging intervention within the workplace setting. Both physical activity and aerobic fitness improved. However, at baseline, participants were more active than they perceived themselves to be. Although there is insufficient evidence to draw strong conclusions about the study findings, it would be useful to test this physical activity text messaging intervention in a sedentary sample within a larger workplace intervention study trial conducted over a longer time frame.

## Introduction

Half of all adults in the United States do not meet the level of recommended aerobic physical activity of ≥ 150 minutes/week of moderate-intensity physical activity or ≥ 75 minutes/week of vigorous-intensity physical activity [1,2]. Engaging in the recommended amount of moderate-intensity physical activity contributes to a significantly lower risk of developing chronic diseases [2]. One type of setting that has been used to test different types of physical activity interventions is the workplace [3]. In fact, in the United States, the Centers for Disease Control and Prevention has issued recommendations to be used for implementing physical activity guidelines in the workplace, in part because it is a logical place to implement physical activity interventions [4]. Workplace clinics are now being used to address a variety of health concerns of employees across the income spectrum. Importantly, workplace chronic disease prevention programs are being used to facilitate promotion of employee health [5-7].

Food service workers represent a growing employee sector in the United States, with Hispanic or Latino employees constituting a significant part of this group [8]. As with many workers, food service employees have work schedule constraints that interfere with finding time for routine preventive health behaviors, including physical activity [9]. Many food service workers are low-income wage earners. Household income influences where people live, which, in turn, can define neighborhood environment and walkability [10-12].

With the widespread use of cell phones and text messaging, there is a new generation of technology available for promoting health that may address some of these constraints that are placed on workers. Physical activity is one of the areas of health promotion where promising results are being seen in the form of text messaging intervention research [13-16]. There are now 7 billion cell phones worldwide [17]. In the United States, there are 317 million cell phones [18], and, on average, 81% of cell phone users engage in text messaging. Cell phone use is common across income levels, including among low-income individuals, of whom 84% have a cell phone [19]. Of Americans who use cell phones, Asian Americans text 69% of the time, white non-Hispanics text 79% of the time, black non-Hispanics text 85 % of the time, and Hispanics text 87% of the time [20,21].

To the best of our knowledge, although workplace physical activity interventions have been conducted in the past, no studies that use text messaging with food service workers to increase physical activity have been published [3,13,14]. The purposes of this study were to determine (1) the feasibility (in terms of recruitment, retention, delivery, and satisfaction) of implementing a bilingual 12-week Text4Walking intervention and (2) the effect of the Text4Walking intervention on change in physical activity (self-report questionnaire, accelerometer, aerobic fitness) and health status (blood pressure [BP], body mass index, waist circumference) in a food service employee population.

## Methods

### Design

This pilot research was conducted using a 1-group 12-week pre- and posttest design.

### Setting and Sample

Participants were recruited from a food service company site located in Chicago, Illinois, the United States. At this food service company, nurse practitioners provide preventative, acute, and chronic health care in a workplace clinic. This food service company is a catering facility that prepares food for retail customers and employs an average of 400 employees, depending on the season. The employees at this company work in either nonadministrative positions, including food preparation, transportation, and facilities management, or as part of the administrative team. The majority of the nonadministrative food preparation workers perform their duties in assembly line-like environments where they repetitively prepare the same types of food for the same retail customer. The company is open 24 hours a day, 7 days a week, every day of the year. To effectively recruit participants from each shift, the research team members covered several 4-hour blocks of time during the week at different times of the day.

For the purposes of this pilot study, a sample size of 32 was needed in order to obtain a power of 0.80. This was determined by using an effect size of 0.72 for physical activity obtained by calculating the mean of effect sizes found in a recent physical activity text message systematic review [13]. With a power of .80, given this effect size and a 1-tailed alpha of 0.05, a sample size of 26 was needed. Oversampling by 20% was done because of the possibility of attrition, resulting in a sample size of 32.

Participants were eligible for this study if they met the following inclusion criteria: (1) currently an employee at the study site; (2) aged 30 to 65 years; (3) able to speak and read English and/or Spanish (by self-report); (4) own a cell phone with texting capability; (5) were sedentary, which is self-reporting as engaging in 20 minutes of vigorous or 30 minutes of moderate physical activity <3 times a week [2]; (6) being without a disability that inhibits walking as determined by the PAR-Q (Physical Activity Readiness Questionnaire) & You [22]; and (7) had no major signs or symptoms of pulmonary or cardiovascular disease, or a systolic BP of >160 mm Hg, or a diastolic BP of >100 mm Hg [23,24]. The age range was chosen because physical activity levels decrease as people approach and enter middle age [1]. The researchers were interested in how participants perceived their physical activity levels, therefore choosing to base inclusion criteria on the initial self-report of physical activity, even if baseline data showed more active physical activity levels than reported by the participant initially.

### Intervention

Using focus groups, the Text4Walking research team developed a database of motivational physical activity text messages in English [25]. Hispanic or Latino adults compose 16.4% of the total number of employed persons in the United States and 24.8% of all adults who are employed in the food service industry [8]. Many Hispanic or Latino adults speak Spanish as their primary language [26]. The Text4Walking team translated the physical activity text messages into Spanish and validated those text messages with a Hispanic focus group [27]. This study used both the English and Spanish text messages (see Multimedia Appendix 1).

A Physical Activity Health Promotion Framework adapted from Pender’s Health Promotion Model [28] was used to guide the Text4Walking intervention. Physical activity text messages were designed to increase perceived benefits of and to decrease perceived barriers to physical activity. To increase commitment to a plan of action, the researchers asked each participant to take part in a 15-minute individual orientation session provided by bilingual research assistants that included a physical activity prescription and strategies for committing to a plan of action to engage in physical activity.

The 12-week goal for each person was to reach a total of 3000 steps (daily) more than their baseline steps. An increase of this amount approximates to 30 minutes of moderate-intensity physical activity daily. This facilitates an individual’s ability to obtain the recommended 150 moderate-intensity physical activity minutes per week [2]. To determine an individualized physical activity prescription, baseline steps were obtained from a pedometer that was taped (no data shown) and worn the week before starting the intervention [29], and a mean of pedometer steps was determined by dividing the days that the pedometer was worn by the total number of steps taken [30]. Each week participants needed to increase their daily step total by 300 steps. Adding 300 daily steps each week to the previous week’s goal, over a period of 10 weeks, allowed participants to achieve the goal of adding 3000 daily steps more than their baseline steps. Their weekly goals were written out for them in the form of a physical activity prescription. Participants were provided with an accelerometer to measure their steps throughout the 12 weeks of the study and given instructions on how to wear and use it. Although participants were encouraged to wear their accelerometers every day, there was no way to enforce that.

Text messaging was used to deliver physical activity text messages and to receive step counts from participants. Participants were told that they would receive 3 text messages a week over the following 12 weeks. The decision to send 3 text messages was made based on prior research done by the team, which showed that, overall, participants preferred this number of motivational text messages a week [25]. These physical activity text messages emphasized the benefits received from increasing one’s physical activity and provided tips on overcoming barriers to engaging in physical activity.

The participants were given a list of more than 250 text messages [25,27], from which they chose a total of 36. If they preferred, they could write up to 10 of their own text messages and choose from the text message database for the remainder of their text messages. Participants chose the 3 days and the time of day during the week when they wanted to receive their text messages. In addition to receiving the motivational physical activity text message, every morning they received a text message that asked them to reply to that message with the previous day’s step count. The research team used a Web-accessible software system that hosted and sent the text messages to participants’ cell phones [31].

In addition, participants received 3 motivational calls of 5-10 minutes each during weeks 2, 6, and 10 of the study from 2 master’s prepared research team members who had received training and were provided with an instruction manual [29]. The main purposes of these motivational physical activity calls were to encourage participants to increase their number of daily steps and the intensity of their walking and to remind them to report their daily steps via text messages.

### Measures

All study questionnaires that were not available in Spanish were translated from English into Spanish by bilingual research team members. They were also reviewed and revised as needed and approved by a certified translator. For the Spanish-speaking participants, a bilingual research team member was available on-site to communicate with them in Spanish.

#### Feasibility Measures

Recruitment was measured by obtaining the screening rate and eligibility and ineligibility rates at baseline. Retention was assessed by obtaining the dropout rate over 12 weeks. Delivery was measured by obtaining the number of text messages generated over 12 weeks. Delivery was also measured by assessing the number of motivational phone calls that were recorded as delivered in the tracking software used in the study. Satisfaction [32] was measured using the Physical Activity Text Messaging Satisfaction Tool. This is a 6-question dichotomous researcher-developed tool. The items in this tool were analyzed for content validity.

#### Participant Baseline Personal and Environmental Characteristics

Age, sex, ethnicity, marital status, educational level, size of household, number of children aged less than 18 years, personal and household income, type of job, and the shift worked were collected at baseline for all participants.

The Neighborhood Environment Walkability Scale–Abbreviated (NEWS-A) was used to assess environmental influences of walkability at baseline. Fifteen items from 4 scales in NEWS-A (land use mix–access, street connectivity, infrastructure and safety for walking, and aesthetics) were combined to create an overall targeted walkability score [33]. These items used a Likert scale, 1 (strongly disagree) to 4 (strongly agree), with higher scores indicating high walkability. Test-retest reliability on NEWS-A ranges from 0.58 to 0.80, and criterion validity has been demonstrated [34]. The items used to create the walkability score for this study had a Cronbach alpha of .83.

#### Physical Activity Outcomes

Physical activity measures were obtained using subjective and objective measures at baseline and 12 weeks, because the researchers were interested in examining both perceived and actual measures of physical activity. Also, both a self-report assessment and an accelerometer-based assessment of physical activity were included in the study because either measure by itself engenders some degree of measurement bias. By including 2 measures, it is possible to obtain some convergent validity [35].

The short International Physical Activity Questionnaire (IPAQ) asked participants about the amount of time they had spent engaged in physical activity in the past 7 days. Seven questions were asked about the days per week and hours and minutes per day spent in vigorous and moderate activity, as well as the amount of time spent walking and sitting, and resulted in walking minutes and moderate and vigorous minutes per day of these activities [36]. Strong reliability has been demonstrated with a Spearman ρ as high as .88, and criterion validity has also been demonstrated [36].

Steps were measured using physical activity monitors. The baseline steps were determined by having participants wear a Kenz Lifecorder e-STEP [37]. The intervention steps were measured during the 12 weeks of the study, using a Lifecorder PLUS [38]. As the researchers were interested in measuring steps taken by participants, both pedometers and accelerometers can be used, as they are reliable tools for estimating the number of steps taken daily [39]. Participants were instructed to wear the accelerometer at their waist during waking hours with instructions to report their step count at the end of each day via text messages. The Lifecorder PLUS recorded time-stamped data, total steps, bout steps, and intensity, for 60 days of data [38]. From this accelerometer wear, we assessed mean daily steps in the 12th week of the study. To be included in the weekly analyses, there had to be ≥10 hours of daily wear time available for 3 or more days during the 12th week. If data were unavailable for that, we accepted data from the 11th week as well. For accelerometer data, an average was taken of the days with acceptable wear time. We also calculated 10-minute bouts of moderate- or vigorous-intensity data [40,41].

To determine aerobic fitness, a 2-minute step test was conducted at baseline and 12 weeks. After warming up, participants marched in place to a minimum knee-stepping height for 2 minutes. This stepping height was individually determined as the level even with the midway point between the iliac crest and the knee [42]. This test is correlated with the Rockport 1-mile walk (*r*=.75) and has concurrent validity with age [43].

#### Health Status Outcomes

Blood pressure was measured at baseline and 12 weeks, following the National High Blood Pressure Education Program guidelines [44-46]. Body mass index and waist circumference were assessed at baseline and 12 weeks [47]. Height was measured using a stadiometer and reported to the nearest 0.25 inch. Weight was measured with participants wearing light clothing and no shoes, using a Seca Robusta 813 high capacity digital scale and reported to the nearest 0.20 pound [48]. Waist circumference was measured using a flexible vinyl tape [49].

### Data Collection Procedures

The study was approved by the University Institutional Review Board [50]. Initial screening was done in person. A research assistant then scheduled interested employees for further screening, which took place before or after work or during a break at the work site health clinic. Employees could complete this assessment, which took an hour or less, in one visit or in multiple visits. Employees were given further explanation of the study, informed consent was obtained, and they had their height, weight, and BP taken. They also completed an aerobic fitness test. If employees had health problems that would impact modest physical activity, they were referred to the study nurse practitioner who reviewed the findings. If needed, the employee was then referred to the workplace health clinic nurse practitioner or their own health care provider to determine if they were eligible or not for the study [24]. Eligible employees then received the study questionnaires and chose their text messages from the data bank. In addition, they were given a blinded pedometer to wear for 7 days and a date and time to return for their study orientation. The study was conducted over a period of 9 months in 2014.

At the study orientation, participants were given their physical activity prescription based on baseline pedometer findings, instructed that they would receive 3 motivational calls throughout the study, and taught how to use the accelerometer Lifecorder PLUS to obtain their daily steps and text them in response to a text message reminder. They were given a participant manual that provided instructions on using their accelerometer and information on receiving and sending text messages. Participants were asked to return at week 7 to have their accelerometer data downloaded. Fresh batteries were provided as needed, and an appointment time was provided for their 12-week final assessment.

At the end of the 12 weeks the participants returned the study accelerometer and completed study questionnaires and health assessments. At both baseline and 12 weeks, participants were given US $15 for completing the assessment and step test, US $10 for completing the study questionnaires, and a US $15 stipend to compensate them for using their text message cell phone service. As a gift, they were given an Omron HJ-112 Premium Pedometer [51] at the completion of the final assessment.

### Data Analysis

All data analyses were performed using SPSS version 19.0 and R 3.0 [40,52]. Because this was a pilot study, any significant findings should be viewed as exploratory. The tables are made up of either means and standard deviations or frequency distributions of the pertinent measures collected in this study. Paired *t* tests were conducted to examine change over time in the sample. For measures with significant skew, a Friedman’s analysis of variance of ranks was also performed.

## Results

### Feasibility

#### Recruitment and Retention

**Figure 1:**
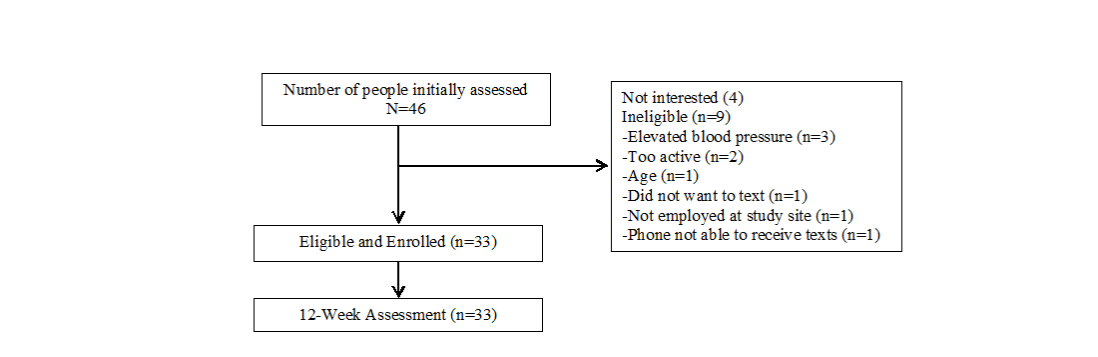
Participant Flow and Study Design of the Text4Walking Intervention with Food Service Employees.

Over a 3-month time period, 46 employees responded to the recruitment strategies and were screened for eligibility (see Figure 1). Further, 4 of them were not interested, and 9 were not eligible. Enrollment was closed once enough participants were enrolled to obtain the anticipated number needed. At the end of recruitment, a total of 33 eligible employees were enrolled in the study, and they all completed it.

#### Delivery

A total of 1188 text messages (33 participants × 3 calls/week × 12 weeks) were generated. Five of the 33 participants chose to develop their own messages; 2 of them created 6 messages each, and 3 of them created 10 messages each. In the study, 1152 text messages were sent to the participants, resulting in a 97% expected delivery rate of text messages, and a mean text messaging rate of 2.9 out of a possible 3 text messages per week. A small number of text messages were not delivered because of unreported changes in participant phone numbers. However, a delivery log was checked weekly by the research team, and phone numbers were updated as soon as possible. The 33 participants received a mean of 1.7 out of a possible 3 study calls. Sixty-one percent (20/33) received the week 2 call, 58% (19/33) the week 6 call, and 46% (15/33) the week 10 call. Three attempts were made to reach the participants for each of the study calls. It is unclear why participants chose not to answer telephone calls.

#### Satisfaction

In general, participants were satisfied with the text message delivery of the intervention. The majority, 91% (30/33), said that they read the text messages. Eighty-eight percent (29/33) thought that 3 text messages a week were the right amount; however, 4 of the participants would have preferred to receive 5 text messages per week. Again, the vast majority (30/33, 91%) thought that the text messages motivated them to increase their physical activity.

### Demographics, Cell Phone Use, and Walkability

Eleven participants self-identified as being of Hispanic or Latino ethnicity (see Table 1). Of these 11 participants, 8 asked to have the intervention delivered in Spanish. Personal income for 63.3% (19/33) of the participants was US $39,999 or less. This is below the national US personal income mean of US $44,888 for 2013 [53]. The jobs were approximately evenly divided between administrative (n=15) and nonadministrative positions (n=18). The majority of the participants owned a smart phone (30/33, 90.9%). Of these 30 participants, 56.7% (17/30) owned an Android phone and 40.0% (12/30) owned an iPhone. The walkability score was 2.8, which is between 2 (somewhat disagree that the neighborhood is walkable) and 3 (somewhat agree that the neighborhood is walkable) on the Likert scale used for this score.

**Table 1:** Participant demographics and walkability.

Characteristics		Total (N=33)
Age in years, mean (SD) (range)		43.7 (8.4) (31-60)
Gender, n		
	Male	14
	Female	19
Ethnicity, n		
	Hispanic or Latino	11
	Not Hispanic or Latino	22
Race, n		
	African American	14
	White	7
	Asian	1
	Unknown	11
Marital status, n		
	Married/living with partner	19
	Widowed/divorced or separated	5
	Single, never married	9
Education, n		
	Some high school or less	8
	Completed high school	10
	Some college or university	10
	Completed college or university	5
Size of household, mean (SD) (range)		3.8 (1.7) (1-7)
Number of children aged <18 years, mean (SD) (range)		1.3 (1.5) (0-4)
Shift, n		
	First shift	27
	Second shift	6
Targeted walkability score, mean (SD) (range)		2.8 (0.3) (1-4)

### Physical Activity Behaviors and Health Status

The IPAQ results demonstrated significant increases in walking minutes per day (see Table 2). However, for walking, moderate, and vigorous minutes there were 0-minute scores for some of the participants. For walking minutes, which meant walking for at least 10 minutes, 7 participants reported 0 minutes at week 1, and 5 participants reported 0 minutes at week 12. For moderate minutes, 14 participants reported 0 minutes at week 1, and 6 participants reported 0 minutes at week 12. Finally, for vigorous minutes, 16 participants reported 0 minutes at week 1, and 11 participants reported 0 minutes at week 12.

For physical activity measured with an accelerometer, there was also a significant improvement in steps walked from baseline to 12 weeks. There was significant skew associated with these data, and, as a consequence, we also conducted a Friedman’s analysis of variance of ranks. When we did so, the *P* value increased from less than .001 to .07. In our study, the effect size was 0.41 for the accelerometer steps. In addition, participants had a significant improvement in their 2-minute step test, suggesting improved aerobic fitness [43].

The mean baseline BP results of the sample are within acceptable ranges for normal BP (Table 2) [44]. Two-thirds (22/33, 66.7%) of the participants were overweight or obese [1]. The average baseline waist circumference for women was above the recommended 35 inches for women, while the average baseline waist circumference for men was below the recommended 40 inches for men [54].

**Table 2:** Physical activity and health status.

Physical activity and health status measures		Baseline week 1	12 weeks	Paired *t* test	Degrees of freedom	*P* value
		Mean (SD) (range)	Mean (SD) (range)			
**IPAQ**^a,b^ (N=33)						
	Walking minutes (minutes/day, on days walked at least 10 minutes)	101.67 (137.88) (0-480)	182.33 (218.64) (0-660)	2.85	32	.008
	Moderate minutes (minutes/day, on days engaged in moderate physical activity)	85.30 (141.52) (0-480)	131.06 (167.62) (0-600)	1.67	32	.105
	Vigorous minutes (minutes/day, on days engaged in vigorous physical activity)	64.64 (131.50) (0-540)	137.42 (205.39) (0-705)	1.95	32	.060
**Steps** (N=22)		10,416 (5097) (3000-19,449)^c^	12,540 (5149) (2773-24,173)^d^	2.59	21	.017
**Intensity data**						
	Minutes of moderate-to-vigorous physical activity per day in 10-minute bouts (N=31)	—	10.5 (12.5) (0-46.9)	—	—	—
	Fitness test (N=33)	87.28 (18.24) (45-123)	96.69 (16.10) (64-121)	4.56	31	<.001
**Health status**						
Systolic BP^a^, mm Hg		120 (12) (99-141)	118 (15) (89-153)	0.96	32	.343
Diastolic BP, mm Hg		76 (10) (57-93)	76 (13) (52-109)	0.24	32	.811
BMI^a^, kg/m^2^		29.29 (6.43) (20.01-51.71)	29.08 (6.66) (19.26-52.12)	1.50	32	.142
	Normal weight (18.5-24.9), n (%)	11 (33.3)	—			—
	Overweight (25-29.9), n (%)	8 (24.2)	—			—
	Obese I, II, III (>30), n (%)	14 (42.4)	—			—
Waist circumference, inches		36.95 (5.19) (26.75-50.00)	37.27 (5.43) (26.00-48.00)	1.12	32	.273
	Female (n=19)	36.36 (4.89) (26.75-46.00)	36.51 (5.19) (26.00-45.50)			—
	Female, >35 inches, n (%)	4 (21.1)	4 (21.1)			—
	Male (n=14)	37.75 (5.66) (31.00-50.00)	38.30 (5.78) (31.00-48.00 )			—
	Male, >40 inches, n (%)	4 (28.6)	5 (35.7)			—

^a^IPAQ: International Physical Activity Questionnaire; BP: blood pressure; BMI: body mass index.

^b^IPAQ data had significant skew.

^c^Pedometer data.

^d^Accelerometer data.

## Discussion

### Principal Findings

The research team was able to obtain participants for the study, and bring them back for their postintervention assessment, in the workplace setting. This may be partially attributed to flexibility in recruiting participants at times that were convenient to them, such as the beginnings and ends of 3 daily work shifts. Furthermore, the intervention appealed to a significant Latino work force because all materials were available in both English and Spanish and intervention research assistants were bilingual. The text messages were successfully delivered, and the participants received these text messages. The participants expressed satisfaction with the content and frequency of the text messages. This may be attributable to the fact that participants were allowed to choose their own text messages as well as create their own. The successful delivery of physical activity text messaging intervention was based on the Physical Activity Health Promotion Framework. The physical activity text messages were designed to reduce perceived barriers to physical activity and to increase commitment to a plan of action.

The intervention was endorsed by the work site clinic, which is a valued employee benefit at this workplace. All assessments were held in the familiar workplace health clinic setting. Many employees had already established a health care relationship with the nurse practitioners at the workplace health clinic, setting the stage for a health promotion intervention [5-7]. Using workplaces to deliver health promotion interventions is important because participants who might otherwise not enroll in an intervention may do so because of the ease of enrolling at their place of work.

One strength of the study was that, even though participants began with a fairly high baseline step count, they significantly increased their step count by more than 2000 steps during this 12-week intervention. They also significantly increased their daily walking minutes and improved their aerobic fitness. In general, participants tend to overestimate the amount of physical activity they engage in when they self-report [55]. It is likely that this occurred in this study as well because the accelerometer steps were somewhat lower than expected based on the participants’ self-reports of physical activity. However, with both measures, participants showed a marked increase in the amount of physical activity from baseline to week 12.

Six studies [56-61] were identified as similar to this study. These six studies examined physical activity as a primary outcome and used text messaging as the only method of motivation for participants. Effect sizes (Cohen’s *d*) for these six studies ranged from 0.29 to 0.64, with a mean of 0.42 [62]. These outcomes were similar to the effect size we found in our study.

One paradoxical finding of the study was that participants who described themselves as sedentary were found to be taking more than 10,000 steps a day when activity was measured with an accelerometer. On the basis of the findings of this pilot study, we will add an objective physical measure when recruiting participants for future studies. Therefore, even if participants self-declare as sedentary, we will have the objective data that is necessary to determine if this is in fact the case.

Another strength of the study was allowing the participants to provide individualized input into delivery of the text messages. This included choosing if they wanted the text messages delivered in English or Spanish and the best days of the week and times of day to receive the text messages. Participants also had an opportunity to create their own text messages. Allowing for participant input into delivery of text messages is similar to other successful physical activity text message studies [57,58,60].

### Comparison With Prior Work

Participants’ mean baseline number of daily steps, 10,416, was considerably higher than the US adult mean of 5117 steps per day [63]. This was despite participants’ self-reporting that they did not engage in 20 minutes of vigorous or 30 minutes of moderate physical activity ≥3 times a week. Workplace employees in a food service job quite likely take more steps during the day than the average US adult does, in part owing to the nature of their jobs. Food service employees stand and walk during their work shifts and may cover fairly large distances while walking in a warehouse setting [64].

### Future Direction

Future studies should include a larger sample as well as a longer intervention phase followed by a maintenance phase to assess the effectiveness of the intervention. In order to test the effectiveness of this intervention, this study should be done with a sedentary sample, using a control group, and a substantially larger number of participants. The length of the study needs to also be increased. Although other health promotion text messaging studies have demonstrated positive outcomes at 12 weeks [65,66], one study noted that lifestyle behavior changes seen at 3 months were not sustained at 6 months [67]. Therefore, using this physical activity text messaging intervention with a less physically active sample may not in and of itself demonstrate the same level of increase as seen in this group of relatively physically active participants, especially if it is used over a longer period of time. It would also be useful to assess maintenance of physical activity after the intervention has ended [68].

### Limitations

The study has several limitations including how physical activity was measured objectively, a potentially biased recruitment process, no control condition, a small sample size, and a modest intervention time frame. Two different physical activity monitors were used at baseline and follow-up steps. Although both monitors are valid for measuring steps, using 2 different monitors may have produced measurement bias. There may have been self-selection bias with more active potential participants volunteering for this small study relative to the size of catchment. Although the researchers intended to recruit a sedentary sample, the actual sample recruited was, on average, an active one. Therefore, it is unknown how an actual sedentary sample would have responded to this intervention. While we were able to retain all of the participants who were recruited, it is possible that the original recruitment process tended to be most successful with the more physically active potential participants. Also, one implication of the no control condition is that it is impossible to determine the impact of text messaging alone in the study.

Although the study had a small sample size, significant findings relative to increased physical activity were discovered [62]. The intervention needs to be further tested with a larger sample using a control condition clinical trial. In addition, although the intervention duration was relatively short (12 weeks), the participants had rather high baseline steps. However, it is unknown if less active adults would increase their physical activity within this same time frame. It is also unknown if the participants in the Text4Walking study would have sustained these results over a longer period of time.

### Conclusions

This paper provides the feasibility and preliminary effectiveness data for a research project. We were able to conduct a motivational physical activity text messaging intervention within the workplace setting. Both physical activity and aerobic fitness improved. However, participants were more active objectively than they perceived themselves to be subjectively. When targeting sedentary participants for a physical activity intervention in the future, it will be critical to assess baseline physical activity with an objective measure to ensure that participants who report they are sedentary are actually part of a low physical activity group. Although there is insufficient evidence to draw strong conclusions about the study’s findings, it would be useful to test this physical activity text messaging intervention in a larger workplace intervention study trial conducted over a longer time frame, in order to confirm that it is effective at increasing physical activity levels in adults.

## Appendix

Multimedia Appendix 1 Physical Activity Text Messages - English and Spanish - Text4Walking.

## Abbreviations

BP: blood pressure
IPAQ: International Physical Activity Questionnaire
NEWS-A: Neighborhood Environment Walkability Scale–Abbreviated
PAR-Q: Physical Activity Readiness Questionnaire
